# Quantitative morphometric and cell-type-specific population analysis of microglia-enriched cultures subcloned to high purity from newborn rat brains

**DOI:** 10.1016/j.ibneur.2021.01.007

**Published:** 2021-02-06

**Authors:** Karolina Dulka, Kálmán Nacsa, Noémi Lajkó, Karoly Gulya

**Affiliations:** Department of Cell Biology and Molecular Medicine, University of Szeged, Szeged, Hungary

**Keywords:** ANOVA, One-way analysis of variance, CNPase, 2′,3′-Cyclic nucleotide 3′-phosphodiesterase, CNS, Central nervous system, DIV, Day(s) in vitro, DMEM, Dulbecco’s Modified Eagle’s Medium, FBS, Fetal bovine serum, FITC, Fluorescein isothiocyanate, GFAP, Glial fibrillary acidic protein, Iba1, Ionized calcium-binding adapter molecule 1, Ki67, Proliferation marker antigen identified by the monoclonal antibody Ki67, PBS, Phosphate buffered saline, PI, Proliferation index, PVP, Polyvinylpyrrolidone, Rpm, Revolutions per minute, RT, Room temperature, S1, S2, Secondary subcultures, S.D., Standard deviation, subDIV, Subcloned day(s) in vitro, T1, T2, Tertiary subcultures, TI, Transformation index, Cell yield, Differential adherence, Immunocytochemistry, Proliferation, Purity of culture, Secondary/tertiary culture

## Abstract

Morphological and functional characterizations of cultured microglia are essential for the improved understanding of their roles in neuronal health and disease. Although some studies (phenotype analysis, phagocytosis) can be carried out in mixed or microglia-enriched cultures, in others (gene expression) pure microglia must be used. If the use of genetically modified microglial cells is not feasible, isolation of resident microglia from nervous tissue must be carried out. In this study, mixed primary cultures were established from the forebrains of newborn rats. Secondary microglia-enriched cultures were then prepared by shaking off these cells from the primary cultures, which were subsequently used to establish tertiary cultures by further shaking off the easily detachable microglia. The composition of these cultures was quantitatively analyzed by immunocytochemistry of microglia-, astrocyte-, oligodendrocyte- and neuron-specific markers to determine yield and purity. Microglia were quantitatively characterized regarding morphological and proliferation aspects. Secondary and tertiary cultures typically exhibited 73.3% ± 17.8% and 93.1% ± 6.0% purity for microglia, respectively, although the total number of microglia in the latter was much smaller. One in seven attempts of culturing the tertiary cultures had ~99% purity for microglia. The overall yield from the number of cells plated at DIV0 to the Iba1-positive microglia in tertiary cultures was ~1%. Astrocytic and neuronal contamination progressively decreased during subcloning, while oligodendrocytes were found sporadically throughout culturing. Although the tertiary microglia cultures had a low yield, they produced consistently high purity for microglia; after validation, such cultures are suitable for purity-sensitive functional screenings (gene/protein expression).

## Highlights

• Microglia can be preferentially shaken off from primary mixed and secondary cultures.
• Secondary cultures contaminated mainly by astrocytes (12–19%) and neurons (2–3%).
• Tertiary cultures had low cell yields (x̄ = 5.1% for T1, x̄ = 2.4% for T2).
• Tertiary cultures had consistently high purity (x̄ = 93.1%; 99% in 1 of 7 attempts).
• Tertiary cultures are suitable for gene or protein expression analyses.

## 1 Introduction

Microglia, the resident immune cells of the central nervous system (CNS), are derived from macrophage-like cells of mesodermal origin and play important roles in both physiological and pathophysiological conditions (Kettenmann et al., 2011, Prinz and Priller, 2014). Microglia migrate to the CNS in the early stages of embryonic development as primitive myeloid progenitor cells and display a ramified morphology at rest (Ginhoux and Jung, 2014, Saijo and Glass, 2011). Inflammation or injury elicits a variety of structural and functional changes that morph these cells from a ramified, resting form to an activated, ameboid type displaying lamellipodia, pseudopodia, filopodia and podosomes (Szabo et al., 2016); this form is also capable of antigen presentation and phagocytosis (Kreutzberg, 1996, Marín-Teva et al., 2004). Activated microglia can exert both beneficial and harmful effects on neurons (Tang and Le, 2016) and astrocytes (Liddelow et al., 2017), as they produce not only neurotrophic factors and anti-inflammatory cytokines, but also potentially neurotoxic mediators, such as neuroinflammatory cytokines, reactive oxygen radicals, nitric oxide and proteases, upon activation (Kraft and Harry, 2011). The amount and quality of such pro- and anti-inflammatory mediators produced by microglia affect the development and progression of neuropathological conditions (Prinz and Priller, 2014, Yao et al., 2013, Liddelow et al., 2017). The rapid response of microglia to inflammatory cues and injury can be studied in vitro and may provide important information regarding the mechanisms underlying neuroinflammation, oxidative stress and the development of neurodegenerative disorders, leading to more effective treatments using microglia as a novel and specific therapeutic target (Gresa-Arribas et al., 2012, Kata et al., 2016, Kata et al., 2017).

In addition to the recently developed protocols that use induced pluripotent stem cells to derivate microglial-like cells (Muffat et al., 2016, Speicher et al., 2019), many conventional cell culture techniques are still available for isolating and culturing microglia from several species (Olah et al., 2012, Szabo and Gulya, 2013, Yip et al., 2009). These cultures are mostly derived from embryonic (Hassan et al., 1991, Gingras et al., 2007, Szabo and Gulya, 2013), newborn or perinatal rodent brains (Ni and Aschner, 2010, Yao et al., 2013), while adult nervous tissues are used less frequently (Moussaud and Draheim, 2010, Pulido-Salgado et al., 2017, Rustenhoven et al., 2016). The methods used for isolating microglia from nervous tissues or for subculturing from a mixed neuronal/glial culture include differential adhesion (shaking) techniques, gradient density centrifugation (Gingras et al., 2007, Moussaud and Draheim, 2010, Jin and Kim, 2015), cell sorting (Ford et al., 1995, Marek et al., 2008) and harvesting microglia from a non-adherent floating cell layer without any shaking at all (Moussaud and Draheim, 2010).

As many of these often-controversial or not fully documented methodological studies used different separation techniques or were carried out without an extensive quantitative analysis of the contaminating cell types present in the cultures, we set out to analyze meticulously our differential adhesion culture method, emphasizing the critical steps that are necessary for the establishment of highly enriched microglial cultures; we also aimed to catalog all of the major contaminating cell types. Immunocytochemistry was applied to the characterization of secondary and tertiary cultures using specific antibodies for microglial, neuronal, astrocyte and oligodendrocyte markers. Morphometric characterization on binary (digital) silhouettes of the microglia collected from different subcultures was used to analyze their area, perimeter and transformation index. Cell proliferation was assessed using the anti-Ki67 antibody, which was raised against the proliferation marker antigen Ki67. Such detailed description of microglial cell cultures established from neonatal rats is unique in the literature.

## 2 Experimental procedures

### 2.1 Animals

All animal experiments were carried out in strict compliance with the European Council Directive (86/609/EEC) and EC regulations (O.J. of EC No. L 358/1, 18/12/1986) regarding the care and use of laboratory animals for experimental procedures, and followed the relevant Hungarian and local legislation requirements. The experimental protocols were approved by the Institutional Animal Welfare Committee of the University of Szeged (II./1131/2018). The pregnant Sprague–Dawley rats (190–210 g) were kept under standard housing conditions and fed ad libitum. Seven breeding runs (with 8–10 pregnant rats in each) provided the litters (6–14 pups from each mother) from which independent cell culture experiments were run to establish the primary and derived cultures.

### 2.2 Antibodies

The antibodies used in our studies are listed in Table 1. For the characterization of the microglial cells, we used an antibody against the ionized calcium-binding adapter molecule 1 (Iba1) (Imai et al., 1996). The anti-glial fibrillary acidic protein (GFAP) antibody (Guillemin et al., 1997), the anti-β tubulin III (Banerjee et al., 1990) antibody and the anti-2′,3′-cyclic nucleotide 3′-phosphodiesterase (CNPase) antibody (Zhang et al., 2010) were used to detect astrocytes, neurons and oligodendrocytes, respectively. The anti-Ki67 antibody was used to detect proliferating cells (Szabo et al., 2016); the Ki67 nuclear protein is expressed in all active phases of the cell cycle, i.e., from the late G1 phase through the end of the M phase (Sobecki et al., 2016).

**Table 1:** Primary and secondary antibodies used in immunocytochemistry.

Primary antibody, abbreviated name	Primary antibody, full name	Final dilution	Company	Secondary antibody with fluorochrome, full name	Final dilution	Company
Iba1	Rabbit anti-Iba1 polycl. ab.	1/500	Wako, Osaka, Japan	Alexa Fluor 488 or 568 goat anti-rabbit	1/1000	Invitrogen, Carlsbad, CA, USA
β tubulin III	Mouse anti-tubulin, β-III, monocl. ab., clone TU-20	1/200	Abcam, Cambridge, England	Alexa Fluor 568 goat anti-mouse	1/1000	Invitrogen, Carlsbad, CA, USA
GFAP	Chicken anti-GFAP	1/200	Abcam, Cambridge, England	FITC-rabbit anti-chicken/turkey	1/250	Invitrogen, Carlsbad, CA, USA
CNPase	Mouse anti-CNPase, monocl. ab., clone 11–5B	1/200	Abcam, Cambridge, England	Alexa Fluor 568 goat anti-mouse	1/1000	Invitrogen, Carlsbad, CA, USA
Ki67	Mouse anti-Ki67, monocl. ab., clone 8D5	1/50	Cell Signaling Technology, Leiden, NL	Alexa Fluor 488 goat anti-mouse	1/1000	Invitrogen, Carlsbad, CA, USA

### 2.3 Preparation of primary mixed cultures from cerebral tissue

Mixed primary neuron/glia cultures were established from newborn rats. Briefly, animals of both sexes were decapitated and the cerebra were removed and cleared from the meninges. The forebrains were minced with scissors and incubated in 9 ml of Dulbecco’s Modified Eagle’s Medium (DMEM; Invitrogen, Carlsbad, CA, USA) containing 1 g/l of d-glucose, 110 mg/l of Na-pyruvate, 4 mM l-glutamine, 3.7 g/l of NaHCO_3_, 10,000 U/ml of penicillin G, 10 mg/ml of streptomycin sulfate and 25 μg/ml of amphotericin B, and supplemented with 0.25% trypsin (Invitrogen) for 10 min at 37 °C, followed by centrifugation at 1000×*g* for 10 min at room temperature (RT) (Szabo and Gulya, 2013). The pellet was resuspended in 10 ml DMEM containing 10% heat-inactivated fetal bovine serum (FBS; Invitrogen) and passed through a sterile filter (100 µm pore size; Greiner Bio-One Hungary Kft., Mosonmagyaróvár, Hungary), to eliminate tissue fragments that resisted dissociation. The filtered cell suspension was centrifuged for 10 min at 1000×*g* at RT and the pellet was resuspended in 5 ml DMEM/10% FBS, after which the primary mixed cells were seeded in the same medium (DIV0) either on poly-l-lysine-coated culture flasks (75 cm^2^; 10^7^ cells/flask) or coverslips (15 ×15 mm; 2 ×10^5^ cells/coverslip). Primary mixed cells seeded on coverslips were used for immunocytochemical comparisons with further subcultures. The cultures were maintained at 37 °C in a humidified air atmosphere supplemented with 5% CO_2_. The medium was changed the next day and then in every 3 days. Unless stated otherwise, the reagents were purchased from Sigma (St. Louis, MO, USA).

### 2.4 Preparation of secondary and tertiary cell cultures

**Fig. 1:**
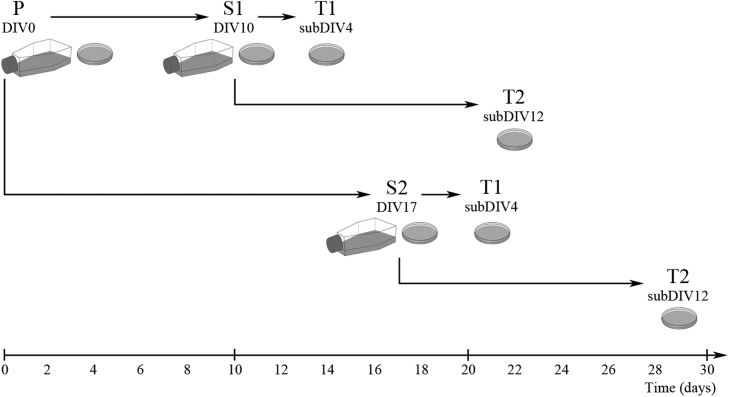
Preparation of microglia-enriched secondary and tertiary cultures from mixed primary cultures. Mixed primary cultures (P, DIV0) were prepared as described in the Experimental procedures. After shaking the cultures, the supernatant was collected. The process was pre-experimentally optimized at 37 °C, 120 rpm for 20 min. Isolated cells were seeded either in Petri dishes or in culture flasks (S1, DIV10; S2, DIV17). Tertiary cultures were subcloned by further shaking the secondary cultures at 37 °C, 150 rpm for 20 min (T1, subDIV4; T2, subDIV12). The primary and secondary cultures were shaken twice. The composition of the secondary and tertiary cultures was then analyzed via quantitative immunocytochemistry using cell-specific markers.

The preparation of secondary and tertiary cultures from mixed primary forebrain cultures is depicted in Fig. 1. After 10 and 17 days of culture (DIV10 and DIV17), microglial cells in the primary cultures were either visualized by immunocytochemistry or shaken off using a platform shaker (120 rpm for 20 min) at 37 °C. During the first and second shaking procedures, at DIV10 and DIV17, respectively, the microglia of the primary cultures were detached from the surface of the poly-l-lysine-coated culture flask. Microglia were collected from the supernatant by centrifugation (3000×*g* for 8 min at RT), resuspended in 4 ml of DMEM/10% FBS and seeded in the same medium on poly-l-lysine-coated culture flasks (75 cm^2^; 10^7^ cells/flask) or coverslips (15 ×15 mm; 2 ×10^5^ cells/coverslip). After the cells were allowed to adhere to the surface for 30 min, the supernatant containing any floating cells was carefully removed and cell culture medium (DMEM/10% FBS) was added to the cells. These cultures were designated as S1 and S2 subclones (from DIV10 and DIV17, respectively). On the fourth and twelfth day of subcloning (subDIV4 and subDIV12), the tertiary microglial cells were subcloned from the secondary cultures that were maintained in poly-l-lysine-coated culture flasks by shaking the cultures at 150 rpm in a platform shaker for 20 min at 37 °C. Microglia were collected from the supernatant by centrifugation at 3000×*g* for 8 min at RT. The pellet was resuspended in 2 ml of DMEM/10% FBS. The number of collected cells was determined in a Bürker chamber after trypan blue staining. The cells were then plated on poly-l-lysine-coated coverslips, for immunocytochemistry. These cultures were designated as T1 and T2 subclones (from subDIV4 and subDIV12, respectively). Primary mixed cells seeded on coverslips were fixed on DIV14, while secondary (S1, S2) and tertiary (T1, T2) cultures were fixed on the next day of subculture in 0.05 M PBS (pH 7.4 at RT) containing 4% formaldehyde for 10 min at RT and stored at −20 °C until use.

### 2.5 Immunocytochemistry

Multicolor immunofluorescence staining was performed as described (Kata et al., 2016, Szabo and Gulya, 2013). Fixed primary, secondary and tertiary cultures on coverslips were rinsed three times for 5 min each in 0.05 M PBS. After permeabilization and blocking of the nonspecific sites for 30 min at 37 °C in 0.05 M PBS containing 5% normal goat serum (Sigma), 1% heat-inactivated bovine serum albumin (Sigma) and 0.05% Triton X-100, the cells on the coverslips were incubated overnight at 4 °C with the appropriate microglia-, neuron-, astrocyte- or oligodendrocyte-specific primary antibody, as well as with the proliferation-specific anti-Ki67 antibody (Table 1). The cultured cells were then washed four times for 10 min each at RT in 0.05 M PBS and incubated without Triton X-100 with the appropriate Alexa Fluor fluorochrome- or fluorescein isothiocyanate (FITC)-conjugated secondary antibody (Table 1) in the dark for 3 h at RT. The cells on the coverslip were washed four times for 10 min each in 0.05 M PBS at RT, and the cell nuclei were stained in a 0.05 M PBS solution containing 1 mg/ml polyvinylpyrrolidone (PBS-PVP) and 0.5 μl/ml Hoechst 33258 dye (Sigma). Finally, the coverslips were rinsed twice for 5 min each time in PBS-PVP, rinsed in distilled water for 5 min, air dried and mounted on microscope slides in Vectashield mounting medium (Vector Laboratories, Burlingame, CA, USA). To confirm the specificity of the secondary antibodies, omission control experiments (i.e., staining without the primary antibody) were performed. In these experiments, no immunocytochemical signals were observed.

### 2.6 Image analysis and statistics

Digital images were captured by a Leica DMLB epifluorescence microscope using a Leica DFC7000 T CCD camera (Leica Microsystems CMS GmbH, Wetzlar, Germany) and the LAS X Application Suite X (Leica). For the determination of the purity of the microglial cultures, the Hoechst 33258-labeled cell nuclei of Iba1-immunopositive cells were counted on coverslip-cultured samples. For each culture, 55–120 randomly selected microscope fields per coverslip from at least 10 coverslips were counted and analyzed using the computer program ImageJ (version 1.47; developed at the U.S. National Institutes of Health by W. Rasband, available at https://imagej.net/Downloads; Schneider et al., 2012). For the measurement of area (A, in μm^2^), perimeter (P, in μm) and transformation index (TI), Iba1-positive microglial images were converted into binary replicas using thresholding procedures implemented by the ImageJ and Adobe Photoshop CS5.1 software (Adobe Systems, Inc., San Jose, CA, USA), as described in detail by Szabo and Gulya (2013). The TI, which is a measure of differentiated cell morphology, was determined according to Fujita et al. (1996) using the following formula: [perimeter of the cell (μm)]^2^/4π [cell area (µm^2^)]. Color correction of the images was occasionally performed when photomicrographs were prepared for publication. Morphometrical comparisons were made using SigmaPlot (v. 12.3, Systat Software Inc., Chicago, IL, USA) and data were analyzed via Kruskal–Wallis one-way analysis of variance (ANOVA) on ranks followed by Dunn’s method; for proliferation assays, via one-way ANOVA followed by Holm–Sidak’s method for pairwise multiple comparisons of differences between the groups; and for the yield data, via Kruskal-Wallis one way ANOVA on ranks, followed by Tukey's test. Values were presented as the mean ± S.D. and significance was set at *p* < 0.05.

## 3 Results

### 3.1 Repeated shaking decreases the yield but increases the purity of microglia in cultures

**Fig. 2:**
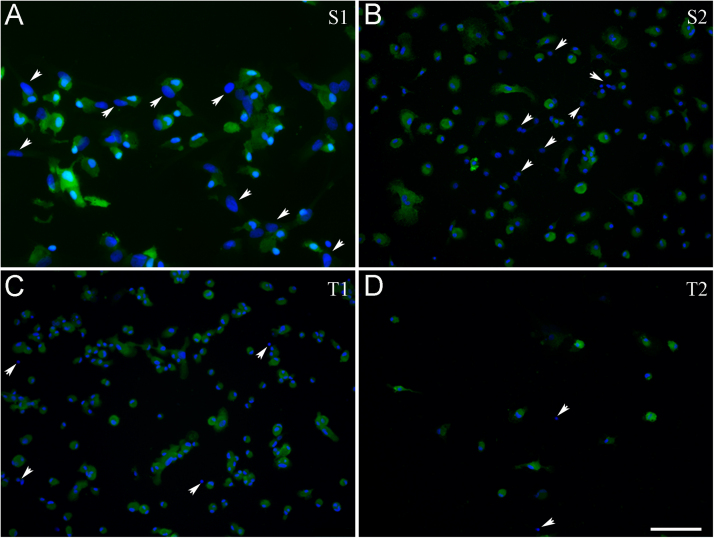
Immunocytochemical localization of microglial cells in secondary and tertiary cultures. The number of Hoechst 33258-labeled cell nuclei (blue) and Iba1-positive (microglial) cells (green) was counted in cultures derived by shaking the primary cultures (S1; A), followed by a second shaking (S2; B); subsequently, the secondary cultures were also shaken twice (T1; C and T2; D). A total of 257,894 cells were counted under a fluorescence microscope using a 20× or 40× objective. Arrows point to cells that do not have Iba1-positive signals. Scale bar: 75 µm.

**Fig. 3:**
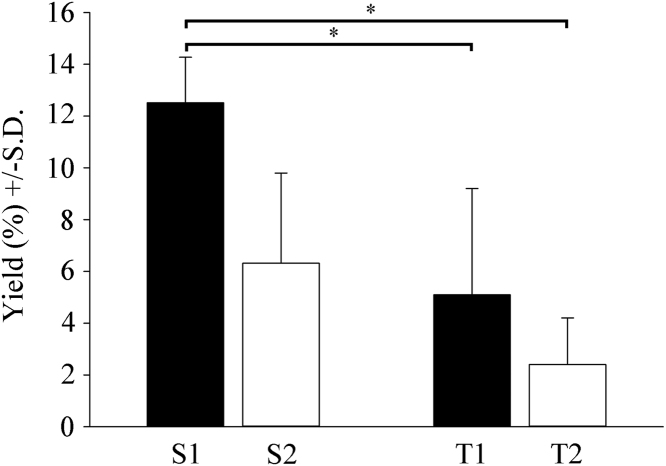
Quantitative analysis of cultured cells in secondary and tertiary cultures. The percentage of the cells collected after shaking the primary and secondary cultures was determined by comparing with the initial number of cells (DIV0 and subDIV0). Despite the use of different (increasing) strengths of shaking of the primary and secondary cultures, we achieved decreasing cell yields in each case. The first shaking of the primary cultures yielded 12.5% ± 1.8% at DIV10 (S1), while the second shaking resulted in a lower yield of 6.3% ± 3.5% at DIV17 (S2). The first shaking of the microglial cells from the secondary cultures resulted in a yield of 5.1% ± 4.1% (T1), while the second shaking of these cells yielded a mere 2.4% ± 1.8% (T2). Newborn rats from seven breeding runs were used to generate seven independent culturing experiments (n = 7). Data are the mean ± S.D. Statistical comparisons were performed using SigmaPlot (v. 12.3, Systat Software Inc., Chicago, IL, USA) and data were analyzed via Kruskal–Wallis one-way analysis of variance on ranks, followed by Tukey's test for statistically significant differences between the groups. * denotes *p* < 0.001.

Primary cultures were shaken twice at 120 rpm for 20 min, first on DIV10, then on DIV17, resulting in the S1 and S2 subcultures, respectively (Fig. 2A and B). These procedures yielded cells that amounted at first to 12.5% ± 1.8%, then 6.3% ± 3.5% of the total cell number of the original primary cultures (DIV0), respectively (Fig. 3). S1 cultures were also shaken twice at 150 rpm for 20 min, resulting in the T1 and T2 subcultures, respectively (Fig. 2C and D), with yields of 5.1% ± 4.1% on subDIV4 and 2.4% ± 1.8% on subDIV12 (calculated from the total cell number at the time of seeding on subDIV0; Fig. 3). In general, repeated shakings resulted in significantly fewer microglial cells that could be harvested (Fig. 3). The overall yield, which was calculated based on the ratio of the number of plated cells at DIV0 to the Iba1-positive microglia in T1 cultures, was ~1%. In our study, secondary cultures resulted 1.6 × 10^6^ ± 1.5 × 10^5^ cells/newborn rat, while tertiary cultures yielded 8 × 10^4^ ± 2 × 10^4^ cells/newborn rat. It is important to emphasize that only one in seven independent cell culture experiments led to the generation of tertiary microglial cultures with a purity of 99% (Table 2).

**Table 2:** Yield and purity of the T1 subcultures in each independent cell culture experiment.

Independent cell culture experiment	Yield of cultured cells in T1 cultures (%)	Purity of T1 cultures for microglia (%)
1.	2.6	93.2
2.	3.2	99.0
3.	2.9	97.2
4.	1.5	80.7
5.	5.4	96.1
6.	6.8	93.9
7.	13.4	91.5
Mean ± S.D.	5.1 ± 4.1	93.1 ± 6.0

### 3.2 Multiple shaking affects microglial morphology

**Fig. 4:**
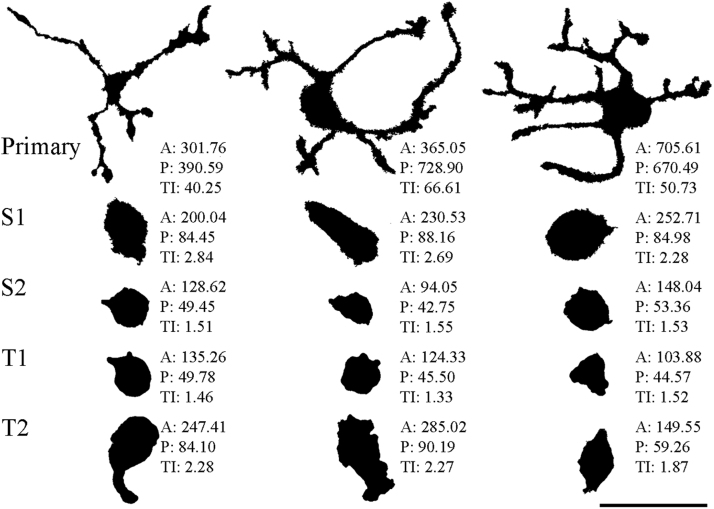
Morphological heterogeneity of microglial cells in different cultures. Iba1-positive microglial cells from the different cultures were photographed, digitized and quantitatively analyzed according to their morphological characteristics. Area (A), perimeter (P) and transformation index (TI) are indicated for each digitized cell. Three representative cells are shown from different microglial cultures. S1: secondary culture established by the first shaking; S2: secondary culture established by the second shaking; T1: tertiary culture established by the first shaking; T2: tertiary culture established by the second shaking. Primary mixed cultures (Primary) were morphologically heterogeneous but exhibited predominantly ramified cell forms with large TI values. Subcultured microglia (S1, S2, T1 and T2) displayed an ameboid form with TI < 3. Note that microglia in T2 cultures had increased cell-surface areas with a longer perimeter and larger TI values compared with secondary or T1 cultures. Scale bar for all silhouettes: 50 µm.

**Fig. 5:**
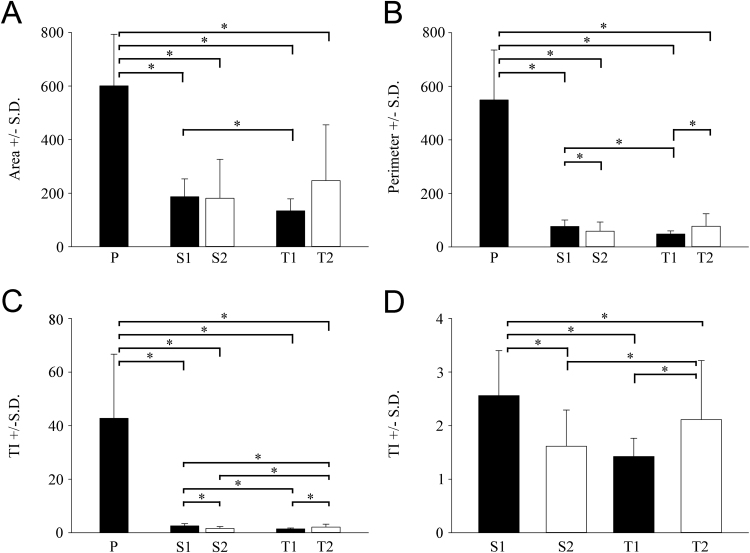
Morphological parameters of microglia in different cultures. Iba1-positive microglia were photographed, digitized and quantitatively analyzed for (A) surface area (μm^2^), (B) perimeter (μm) and TI value (C and D) from different microglial cultures. (C) and (D) are related. Statistical comparisons were performed using SigmaPlot (v. 12.3, Systat Software Inc., Chicago, IL, USA) and data were analyzed via Kruskal–Wallis one-way analysis of variance on ranks, followed by Dunn’s method for pairwise multiple comparison procedures for statistically significant differences between the groups. P: primary mixed neuron/glial culture; S1: secondary culture established by the first shaking; S2: secondary culture established by the second shaking; T1: tertiary culture established by the first shaking; T2: tertiary culture established by the second shaking. Values are presented as the mean ± S.D. Significance was set at *p* < 0.05. * denotes *p* < 0.05.

The morphological features of microglial cells (A, P and TI), were quantitatively analyzed using binary silhouettes on a total of 214 cells (Figs. 4 and 5). In the primary mixed neuron/glia cultures at DIV14, most of the microglial cells were ramified with an average area of 601.1 ± 192.2 µm^2^, a perimeter of 549.0 ± 186.6 µm and a TI value of 42.8 ± 23.9 (Figs. 4 and 5). Microglia in secondary (S1 and S2) and tertiary (T1 and T2) cultures displayed significant morphological differences, as secondary cultures had a larger cell size and shorter projections compared with those in tertiary cultures. The S1 subculture consisted mainly of larger ameboid microglia with small pseudopodia (A = 186.6 ± 66.7 µm^2^, P = 76.7 ± 23.8 µm, TI = 2.6 ± 0.8) (Figs. 4 and 5), while the S2 subculture microglia became more ameboid, with a slightly smaller cell body and without pseudopodia (A = 181.0 ± 145.8 µm^2^, P = 58.9 ± 33.9 µm, TI = 1.6 ± 0.7) (Figs. 4 and 5). Interestingly, while T1 subcultures consisted of microglia with a typical ameboid morphology (A = 134.0 ± 44.9 µm^2^, P = 48.4 ± 11.4 µm, TI = 1.4 ± 0.3), T2 subcultures contained microglia with shapes that varied from mostly small spherical to a few large flattened cells (A = 247.0 ± 207.9 µm^2^, P = 77.4 ± 46.4 µm, TI = 2.1 ± 1.1) (Figs. 4 and 5). Damaged cells and cell debris were also found in T2 cultures, as multiple shakings reduced the survival of microglia because of mechanical stress.

### 3.3 Secondary and tertiary cultures have a different cell-type composition

**Fig. 6:**
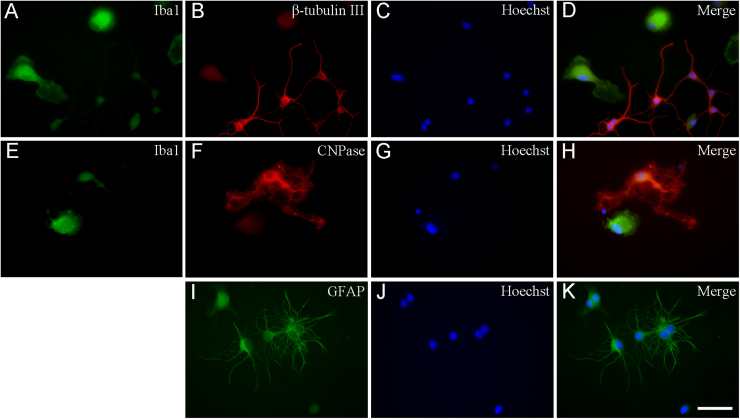
Immunocytochemical localization of microglia, neurons, astrocytes and oligodendrocytes in secondary cell cultures. Different cell types were labeled by the corresponding marker proteins. An anti-Iba1 antibody was used to detect microglia (green in (A) and (E)), an anti-β tubulin III antibody was used to detect neurons (red (B)), an anti-CNPase antibody was used to detect oligodendrocytes (red (F)) and an anti-GFAP antibody was used to detect astrocytes (green (I)). These primary antibodies were recognized by Alexa Fluor or FITC-conjugated secondary antibodies. The cell nuclei were stained with Hoechst 33258 (blue (C), (G), (J)). Merged images are also shown ((D), (H), (K)). Scale bar: 50 µm. (For interpretation of the references to colour in this figure legend, the reader is referred to the web version of this article.)

**Fig. 7:**
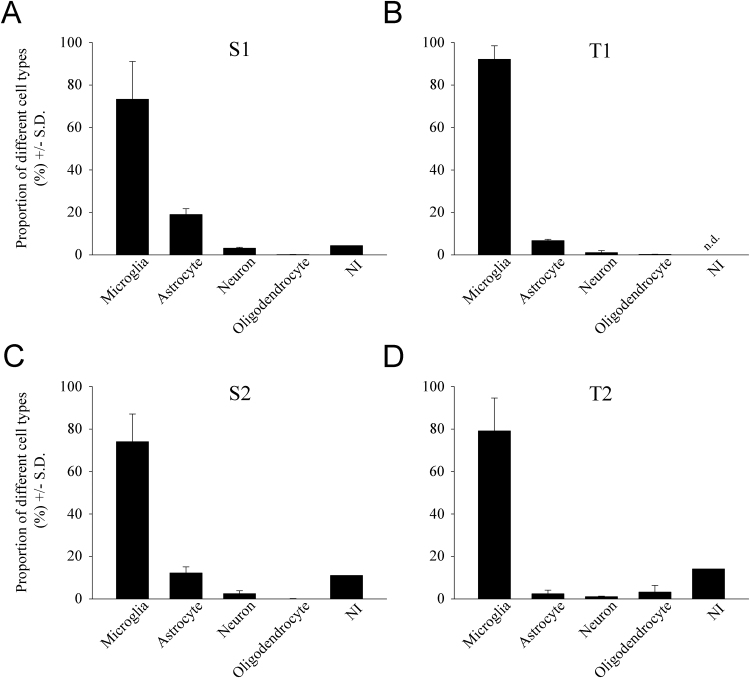
Quantitative analysis of the distribution of different cell types in secondary and tertiary cultures. Microglia-enriched cultures were obtained after the shaking of the primary cultures (S1 = 73.3%, S2 = 74.1%; (A) and (C), respectively). Shaking of the secondary cultures led to tertiary cultures with higher purities (T1 = 93.1%, T2 = 79.3%; (B) and (D), respectively). The number of astrocytes and neurons progressively decreased throughout culturing. Occasionally, a few cells that were not identified (NI) immunocytochemically in this study were also observed in secondary cultures; such cells were exceptionally rare in T1, but were somewhat more numerous in T2 cultures. n.d.: not detected.

Cell-specific markers were used to identify the cells in the cultures by immunocytochemistry (Fig. 6). Secondary cultures were enriched in microglia with a mean purity of 73.3% ± 17.8% (Fig. 7A). In addition to microglia (Fig. 6A and E), a relatively large number of GFAP-immunoreactive astrocytes (19.0% ± 2.7%; Figs. 6I and 7A), a smaller number of β-tubulin III-positive neurons (3.1% ± 0.4%; Figs. 6B and 7A) and a few CNPase-positive oligodendrocytes (0.2% ± 0.1%; Figs. 6F and 7A) were found in these cultures. Approximately 4.4% of the cells in these cultures remained unidentified (Fig. 7A). The second shaking of the primary mixed neuron/glia cultures (S2) produced remarkably similar results: microglia, astrocytes, neurons and oligodendrocytes comprised 74.1% ± 13.1%, 12.3% ± 2.8%, 2.5% ± 1.4% and 0.1% ± 0.1% of the total number of cells, respectively. Unidentified cell types represented about 11% of the total cell population (Fig. 7C).

From the first shaking of the secondary culture, a high purity tertiary microglial culture was prepared, in which almost all cells were Iba1 immunoreactive. For example, T1 cultures contained 93.1% ± 6.0% microglia (with the highest purity being ~99.0%), 6.8% ± 0.6% astrocytes, 1.1% ± 0.9% neurons and 0.2% ± 0.1% oligodendrocytes (Fig. 7B). When secondary cultures were shaken for the second time, the purity of the cultures decreased as the amount of unidentified cell types rose to 14.1%. These T2 cultures contained 79.2% ± 15.5% microglia, 2.4% ± 1.7% astrocytes, 1.1% ± 0.3% neurons and 3.2% ± 3.1% oligodendrocytes (Fig. 7D). Although tertiary cultures produced a low cell yield, they had a consistently high purity, which rendered them suitable for purity-sensitive functional screenings, such as gene or protein expression analyses.

### 3.4 Quantitative analysis of proliferating microglia in secondary and tertiary cultures

**Fig. 8:**
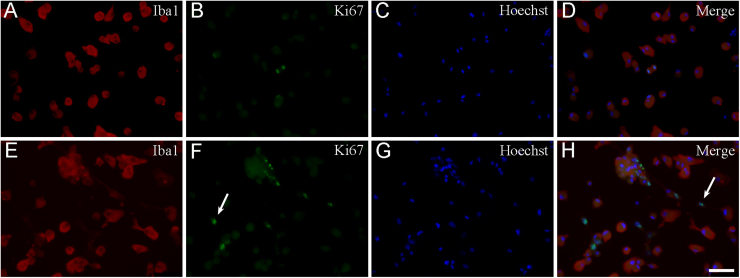
Representative immunocytochemical images of Ki67-positive microglial and non-microglial cell types in secondary cultures. Cultured Iba1-positive microglia (red (A), (E)), proliferating Ki67-positive cells (green (B), (F)) and Hoechst 33258-labeled cell nuclei (blue (C) and (G)) were identified. Merged images are provided in panels (D) and (H). Note that some of the Ki67-positive cell nuclei (arrow in panel (F)) did not belong to Iba1-positive microglia. Scale bar: 75 µm. (For interpretation of the references to colour in this figure legend, the reader is referred to the web version of this article.)

**Fig. 9:**
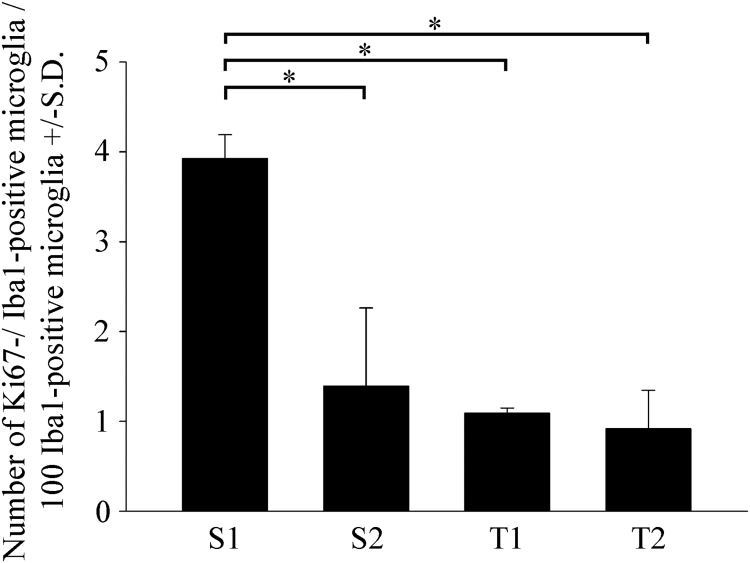
Quantitative analysis of Ki67-positive microglia in secondary and tertiary cultures. Proliferation was measured as a function of Ki67 immunopositivity of the Iba1-immunopositive microglial cells in secondary (S1, S2) and tertiary (T1, T2) cultures, and calculated using the following equation: PI = number of Ki67-positive microglia / total number of Iba1-positive microglia × 100. KI67-positive microglia were counted in 290 microscopic fields of view from three separate cell culture experiments. Statistical comparisons were performed using SigmaPlot (v. 12.3, Systat Software Inc., Chicago, IL, USA) and analyses were carried out via one-way analysis of variance followed by pairwise multiple comparisons (Holm–Sidak method). The results are presented as the mean ± S.D. Significance was set at *p* < 0.05.

To determine the rate of proliferating microglia, double-fluorescence immunocytochemistry was performed using the anti-Ki67 and anti-Iba1-antibodies (Fig. 8), and the proliferation index (PI) was determined (Fig. 9). The PI was defined as the ratio of Ki67-positive/Iba1-positive microglial cell nuclei per the total number of Iba1-positive cells × 100. From a total of 18,000 analyzed cells, 14,634 were Iba1-positive and 411 were Ki67-positive/Iba1-positive microglia across 290 microscopic fields of view. In one experiment, for example, from a total of 2454 analyzed secondary cultured cells, 2149 were Iba1-positive (87.6% purity) and 85 were Ki67-positive/Iba1-positive microglia (PI = 3.9%) across 30 microscopic fields of view. According to Ki67 immunocytochemistry (Fig. 9), the number of proliferating microglia in the S1 cultures, on average, was about 3-fold larger (PI = 3.9% ± 0.3%) than those detected in the S2 (PI = 1.4% ± 0.9%) or the tertiary cultures (PI = 1.1% ± 0.1%; PI = 0.9% ± 0.4% in T1 and T2, respectively), proving that the shakings affected cell composition and proliferation characteristics of the cultures. Both quantitative morphological and Ki67 immunocytochemical studies indicated that the derived microglia were ameboid microglia capable of proliferation.

## 4 Discussion

Microglial cultures derived from primary mixed neuronal/glial cultures are useful tools for studying fundamental morphological and functional characteristics of microglia, e.g., motility, secretory mechanisms, activation states, etc. (Stansley et al., 2012). Culturing microglia is a time-consuming and meticulous process that ultimately results in a low cellular yield with a variety of contaminating cell types (mainly astrocytes and oligodendrocytes, but also pericytes, fibroblasts, smooth muscle cells and endothelial cells) that degrade the purity of the culture (Saura, 2007).

Several studies exploited differential cellular adhesion to harvest microglia. Some used mild trypsinization (Saura et al., 2003), while others used a varied length and strength of shaking. In addition to shorter agitation times, e.g., 45 min or 3 h (Flode and Combs, 2007, Gingras et al., 2007), more vigorous methods such as 180 rpm for 15 h (Giulian and Baker, 1986) or 150 rpm for 16 h (Hassan et al., 1991) were also used. Other authors used gentle agitation, e.g., shaking the flasks by hand and gently blowing with a pipette (Qin et al., 2012) or gently banging on the side and tapping the flasks at a speed of 45 rpm for about 9–12 min (Ni and Aschner, 2010). Typical protocols reported enzymatic digestion using differential adhesion steps with the shaking off of non-adherent cells after 2 or 24 h (Yip et al., 2009) or just 10 min (Gingras et al., 2007). While some researchers used a microglia enrichment step only once (Saura et al., 2003, Rustenhoven et al., 2016), others isolated microglia repeatedly from the same primary culture: 3 times after 7 days (Flode and Combs, 2007), 3 times after 8–10 days with passage (Qin et al., 2012), 2 times after 1 month (Gingras et al., 2007) and twice a week from the supernatant without shaking up to 32 days (Moussaud and Draheim, 2010). The purity of these microglia-enriched cultures was typically high and was accompanied by a low yield. Complex protocols with mechanical and enzymatic dissociation followed first by density separation, then immunomagnetic separation resulted in a low yield (7.5 × 10^5^ cells/mouse) but high purity (98%) even from an adult brain (de Haas et al., 2007). Qin et al. (2012) reported one of the best yields, an average of 7 × 10^6^ cells/neonatal rat, after 3 shakings with passages, while Gordon et al. (2011) used a column-free magnetic separation technique with tetrameric antibody complexes to produce 3 × 10^6^ microglia/mouse pups with 97% of purity after only one shaking at 120 rpm for 2.5 h. Yield can be increased using GM-CSF treatment (Gingras et al., 2007, Moussaud and Draheim, 2010). Despite the low yield, the most popular and widely used methods are based on the differential adherence isolation of the different cell types, mainly because they offer simple techniques and low cost. In addition to the harvesting techniques, other factors (different donor species, age, tissue type, coated or uncoated culture vessels, etc.) further contributed to the observed differences in yield and cell number.

Our previous studies demonstrated that the number of microglia constantly increased from immediately after seeding throughout the entire cell culture period (Szabo and Gulya, 2013), which allowed the shaking off of microglial cells more than once during culture. Therefore, we harvested microglia from two time points from the primary (DIV10 and DIV17) and secondary (subDIV4 and subDIV12) cultures, which resulted in the S1 and S2 and T1 and T2 subcultures, respectively. These cultures differed both in yield and morphological parameters, as evidenced by proliferation studies and quantitative morphological analyses of the binary silhouettes of the microglia. We found that secondary cultures were enriched (up to 73%) in microglia, and that repeated shaking of the same primary cultures decreased the yield and resulted in a lower microglial yield. Due to their low number, astrocytes will not form a confluent layer to which microglia could attach in secondary cultures, so microglia can easily be shaken off and seeded as a tertiary culture. Those microglia that attach directly to the plastic dish will most likely remain on the surface after shaking; we saw some of these remaining microglia cells under the microscope (data not shown). The low yield in tertiary cultures was probably due to this fact. Nevertheless, tertiary cultures had a consistently high purity. It is important to note that only one out of seven attempts of cell culture resulted in ~99% purity. Immunocytochemical validation of harvested samples using microglial markers allowed us to select cultures to be used in purity-sensitive functional screenings of gene or protein expression (Kata et al., 2016, Kata et al., 2017). Using a similar protocol, we previously provided evidence that microglia in secondary cultures could be immunochallenged by lipopolysaccharide and were shown to behave as expected in functional and gene expression studies (Kata et al., 2016, Kata et al., 2017, Szabo and Gulya, 2013, Szabo et al., 2016). In these studies, phagocytosis, proliferation and protein expression assays, pro- and anti-inflammatory cytokine (IL-1beta, tumor necrosis factor alpha, IL-10) production, as well as cytoskeleton reorganization studies and gene expression assays (testing 122 inflammation-related genes) were employed to test the functionality of in vitro cultured microglia. The present work focused on the purity/yield characteristics of the cultures and cataloged the cell types that make up these populations; we are aware that our method needs further functional studies to characterize microglia in these cultures.

Our secondary microglial cultures were contaminated mainly by astrocytes (12–19%) and, to a lesser degree, by neurons (2–3%) and oligodendrocytes (about 0.2%). Generation of the main cell types in the brain occurs in a temporally distinct yet overlapping pattern (Sauvageot and Stiles, 2002). In rats, neurogenesis peaks between E14 and E18, while astrocytogenesis at P2 (astrocytogenesis starts around E18 in mice) and oligodendrocytogenesis peaks at P14, although oligodendrocyte precursor cells appear earlier (Reemst et al., 2016, Sauvageot and Stiles, 2002). At the time of birth, non-neuronal cells comprise only about 6% of all brain cells in the rat (Bandeira et al., 2009), out of which perhaps 1–2% are microglia. Cellular abundances in newborn tissues are reflected in the composition of the primary cultures from which subsequent cultures are made. Astrocytes, the most contaminating cell type throughout culturing, attach to the surface strongly. Nevertheless, we were able to decrease their contamination in the secondary and tertiary cultures by shaking off the microglia from the astrocytic layer; in fact, this is the main reason we made tertiary cultures. It has to be taken into consideration that, although GFAP is a well-documented astrocyte marker, not all astrocyte subtypes express it at high levels, e.g., protoplasmic astrocytes of the gray matter (Emsley and Macklis, 2006, Miller and Raff, 1984, Reemst et al., 2016, Tabata, 2015). Approximately 4.4% of the cells in the secondary cultures remained unidentified, and some of them could be those protoplasmic astrocytes with low abundance of GFAP.

While the amounts of all contaminating cell types decreased considerably, the largest reduction in tertiary cultures was observed in the number of astrocytes (down to 2.4% ± 1.7% in T2). In fact, in similarly enriched microglial cultures, astrocytes were detected most often. For example, Saura et al. (2003) reported that, in such cultures, 0.2–0.5% of the cells were GFAP-positive astrocytes and that 0.8–1.7% of the cells expressed smooth muscle α actin, which is a marker protein of both pericytes and astrocytes. A similar value (2.4%) was detected for pericytes by Rustenhoven et al. (2016) in highly enriched primary human microglial cultures.

The percentage of oligodendrocytes was typically around 0.5% (Gingras et al., 2007, Giulian and Baker, 1986, Gordon et al., 2011, Ni and Aschner, 2010, Saura et al., 2003, Yip et al., 2009). Other much less frequent cell types are rarely counted. Moussaud and Draheim (2010) looked for the presence of neurons and fibroblasts in a culture containing 98% microglia, although they did not publish numerical data, merely figures derived from flow cytometry. Similarly, Tamashiro et al. (2012) noted only minimal contamination, without specifying the contaminating cell types.

Microglia become ameboid after shaking the cultures. We demonstrated that the cells obtained from the second shaking were more spherical than those obtained from the first shaking of primary cultures, as documented by their decreasing TI values. This morphological response could be attributed to the mechanical stress upon the more ramified microglia that remained attached to the surface in the primary culture. In tertiary cultures, microglia displayed increased TI values in T2 cultures compared with T1 cultures; however, these values remained lower than those detected in secondary cultures, probably as a consequence of the more varied cell sizes and shapes present in T2 cultures. One of the drawbacks of the differential cellular adherence method is that consecutive shaking acts as a mechanical stress that results in progressively fewer surviving cells. Because of such mechanical effects, which are mediated by contact-dependent structures via cytoskeletal remodeling (Yan et al., 2012, Vincent et al., 2012), microglia easily detach from the surface of culture materials. Although many of these mechanisms depend on calmodulin-mediated phenomena (Siddiqui et al., 2012, Szabo et al., 2016), the exact mechanisms underlying the manner in which the cytoskeleton adapts to the shearing forces of shaking and the changes in the expression of adhesion-promoting molecules remain largely unknown.

The establishment of immortalized microglial cell lines facilitated their culture in bulk. In these cultures, the cell population is homogeneous and a higher yield can be generated within a short period (Blasi et al., 1990, Nagai et al., 2005, Stansley et al., 2012). Undoubtedly, these cell lines are very similar both morphologically and functionally to microglia cultured from brain tissue, but the oncogene expressed by these cells could contribute to the altered morphological and functional characteristics (proliferation and adhesion capabilities, gene expression differences, etc.) of these microglia. However, altering the genetic parameters of these cells may not only provoke a non-physiological behavior in the cells or lead to genetic instability, but also potentially hamper the proper screening of these cells for therapeutic intervention. To avoid these complications, ex vivo microglia cultured to high purity (>98% with an acceptable yield) could be better suited for downstream applications that require high purity, such as gene or protein expression. Thus, there will always be reasons to culture microglia from wild-type or genetically altered animals. The ex vivo culturing of microglial cells is also feasible if an analysis of in-house genomic modification of the animals is the goal of the experiment. The current technologies do not require high yield, as even single-cell-based gene expression analysis is widely available (Li et al., 2019, Matcovitch-Natan et al., 2016). Our protocol does not need expensive instrumentation and may abide the test of time especially when larger amount of cells or protein content are necessary for purity-sensitive functional screenings such as ELISA or Western analysis.

## CRediT authorship contributions statement

Conceived and designed the experiments: KG. Performed the experiments: KD, KN, NL. Analyzed the data: KD, KN, NL. Contributed reagents/materials/analysis tools: KG. Wrote the paper: KD, KN, NL, KG. Edited the paper: KG. All authors have read and approved the final manuscript.

## Conflict of Interest

The authors declare that they have no conflict of interest.
